# Synthetic and Functional Engineering of Bacteriophages: Approaches for Tailored Bactericidal, Diagnostic, and Delivery Platforms

**DOI:** 10.3390/molecules30153132

**Published:** 2025-07-25

**Authors:** Ola Alessa, Yoshifumi Aiba, Mahmoud Arbaah, Yuya Hidaka, Shinya Watanabe, Kazuhiko Miyanaga, Dhammika Leshan Wannigama, Longzhu Cui

**Affiliations:** 1 Division of Bacteriology, Department of Infection and Immunity, School of Medicine, Jichi Medical University, Shimotsuke 329-0498, Japan; alessa.ola@jichi.ac.jp (O.A.); y-aiba@jichi.ac.jp (Y.A.); d2222@jichi.ac.jp (M.A.); hidaka.yuya@jichi.ac.jp (Y.H.); swatanabe@jichi.ac.jp (S.W.); miyanaga.kazuhiko@jichi.ac.jp (K.M.); 2 Department of Infectious Diseases and Infection Control, Yamagata Prefectural Central Hospital, Yamagata 990-2292, Japan; leshanwannigama@gmail.com

**Keywords:** bacteriophage engineering, synthetic biology, phage assembly and rebooting, phage recombineering, CRISPR-Cas systems, retron-mediated editing, non-replicative phage, host range expansion, antimicrobial delivery platforms, cell-free TXTL systems

## Abstract

Bacteriophages (phages), the most abundant biological entities on Earth, have long served as both model systems and therapeutic tools. Recent advances in synthetic biology and genetic engineering have revolutionized the capacity to tailor phages with enhanced functionality beyond their natural capabilities. This review outlines the current landscape of synthetic and functional engineering of phages, encompassing both in-vivo and in-vitro strategies. We describe in-vivo approaches such as phage recombineering systems, CRISPR-Cas-assisted editing, and bacterial retron-based methods, as well as synthetic assembly platforms including yeast-based artificial chromosomes, Gibson, Golden Gate, and iPac assemblies. In addition, we explore in-vitro rebooting using TXTL (transcription–translation) systems, which offer a flexible alternative to cell-based rebooting but are less effective for large genomes or structurally complex phages. Special focus is given to the design of customized phages for targeted applications, including host range expansion via receptor-binding protein modifications, delivery of antimicrobial proteins or CRISPR payloads, and the construction of biocontained, non-replicative capsid systems for safe clinical use. Through illustrative examples, we highlight how these technologies enable the transformation of phages into programmable bactericidal agents, precision diagnostic tools, and drug delivery vehicles. Together, these advances establish a powerful foundation for next-generation antimicrobial platforms and synthetic microbiology.

## 1. Introduction

**Figure 1:**
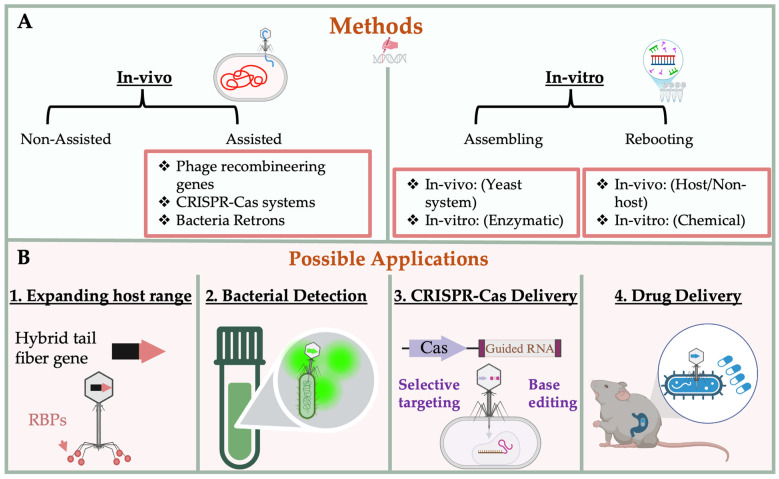
Graphical Abstract of Phage Engineering Strategies and Representative Applications. (**A**) Overview of available phage engineering approaches categorized into in-vivo and in-vitro methods. (**B**) Representative applications of engineered phages, including (1) expanding host range via modification of phage tail fiber proteins to express alternative receptor binding proteins (RBPs); (2) bacterial detection through the incorporation of reporter genes or molecular tags; (3) delivery of CRISPR-Cas systems for targeted bacterial elimination or genome editing; and (4) in situ expression of therapeutic proteins for antimicrobial or modulatory purposes.

The rapid evolution of molecular tools and synthetic biology platforms has transformed bacteriophages from naturally occurring antibacterial agents into versatile and programmable biological systems. Engineered phages now serve as modular platforms for precise host targeting, genetic payload delivery, and antimicrobial intervention. These advances are underpinned by a growing repertoire of in vivo genetic manipulation techniques and in-vitro synthetic assembly and rebooting systems, which collectively enable fine control over phage structure, host range, and functional output. In this review, we contextualize recent developments in phage engineering by tracing their historical origins, outlining the biological principles essential for design, and detailing current methodologies and emerging applications in therapeutic, diagnostic, and delivery technologies (Figure 1).

### 1.1. Historical Background of Bacteriophage Research

Bacteriophages, commonly referred to as phages, are viruses that specifically infect and replicate within bacteria. The first encounter with the therapeutic properties of phages is believed to date back to 1896, when Ernest Hankin reported the discovery of filterable and heat-sensitive antibacterial agents in the Ganges and Yamuna rivers in India, which contributed to the prevention of the spread of *Vibrio cholerae* infections [1,2]. However, phages were first reported independently by Frederick Twort in 1915 [3] and by Félix d’Hérelle in 1917 [4]. These discoveries became known as the “Twort and d’Hérelle phenomena” [5]. Shortly thereafter, the therapeutic potential of phages attracted significant interest from many scientists [6], including d’Hérelle himself, whose efforts culminated in the successful treatment of the first patient in 1919 [7,8]. However, this interest waned with the discovery of antibiotics and remained primarily active in parts of Western Europe and the former Soviet Union [6]. Meanwhile, phages continued to contribute significantly to molecular biology, serving as model systems for elucidating fundamental principles of genetics and recombination. Many of the genetic recombineering techniques and molecular biology tools currently in use were originally discovered or inspired through phage research. These include the demonstration of DNA as the sole carrier of genetic information [9,10], the potential for genetic recombination between closely related DNA molecules (phage crossing) [11], the discovery of transposable elements (phage Mu) [12], the identification of restriction enzymes [13], DNA ligation enzymes (T4 DNA ligase) [14], and notably, the sequencing of the first complete genome belonging to a phage [15]. In other words, phages have played a pivotal role in the development of molecular genetics, laying the groundwork for today’s genetic engineering and synthetic biology.

### 1.2. Modern Applications and the Need for Phage Engineering

Phages are ubiquitous in nature, including the human body [16], with an estimated presence of 10^31^ particles on the planet [17]. Their abundance may exceed that of bacteria in certain ecosystems [18]. Although phages are structurally simple and possess compact genomes, they exhibit remarkable diversity in virion architecture, genetic content, and host specificity. As natural predators of bacteria, phages have emerged as promising alternatives to antibiotics, particularly in the context of rising multidrug-resistant bacterial infections [19,20]. However, therapeutic success often requires case-by-case optimization, and phages frequently showed improved efficacy when used in combination with antibiotics [21].

Limitations such as narrow host ranges, variable lytic activity, and safety concerns have spurred efforts to develop engineered phages with enhanced properties. A major area of research focuses on modifying or replacing tail fiber proteins to redirect phage host specificity [22,23,24,25]. In addition, phages or phage particles have been engineered to perform novel functions, including biofilm degradation [26,27,28], pathogen detection [29,30,31,32], delivery of therapeutic proteins [33], targeted gene disruption via CRISPR-Cas systems [23,34,35,36,37,38], antibacterial peptide delivery [39,40], and bacteria base editing [22].

Moreover, recently, phage structural components, such as the phage capsid [41] and tails [42], have been repurposed as synthetic delivery vehicles, allowing for the development of non-replicative, biocontained phage platforms capable of carrying diverse therapeutic cargos. These advances highlight the growing convergence of molecular virology, genetic engineering, and synthetic biology, opening the door to designing phages not only as antibacterial agents but also as precision tools for diagnostics, gene editing, and therapeutic delivery (Table 1).

## 2. Biological Foundations of Phage Engineering

Before discussing phage engineering methods, it is essential to outline the fundamental biological characteristics of phages, including their structure, life cycles, DNA packaging mechanisms, and host recognition strategies. A thorough understanding of these features is critical for selecting appropriate engineering approaches, as each phage’s genomic organization, packaging mechanism, and host range determinants influence the design and success of genetic or synthetic modifications.

### 2.1. Phage Structure and Morphology

Phages are, in principle, structurally simple, consisting of genetic material encased in a protective protein known as the capsid. Based on their capsid morphology, phages are broadly classified into two categories: tailed and non-tailed [45]. Electron microscopy studies have revealed that approximately 96% of all known phages are tailed and belong to the order *Caudovirales* [46]. Tailed phages are further subdivided into five major families. Three of these—*Myoviridae*, *Ackermannviridae*, and *Herelleviridae*—possess contractile tails and were historically grouped as myophages. The remaining two families—*Siphoviridae* and *Podoviridae*—feature a noncontractile tail, with *Siphoviridae* having long tails and *Podoviridae* having short ones. Well-characterized examples include *Escherichia coli* phages such as T4, P1, and P2 (*Myoviridae*), λ and T5 (*Siphoviridae*), T7, T3, and PhiV10 (*Podoviridae*), and EP75 (*Ackermannviridae*); *Salmonella* phages such as P22 (*Podoviridae*) and S16 (*Myoviridae*); and *Staphylococcus* phages such as phage K (*Myoviridae*), phage SA75 (*Siphoviridae*), and phage Twort (*Herelleviridae*). In contrast, non-tailed phages—such as those belonging to *Inoviridae* (e.g., M13)—may exhibit cubic, filamentous, or pleomorphic morphologies [18]. Phages also vary in genomic composition: their genomes may consist of single-stranded (ss) or double-stranded (ds) DNA or RNA [47]. Genome sizes range from a few kilobases (kb), such as Campylobacter phage C10 (1.4 kb) [48], to over 200 kb in Jumbo phages [49], with megaphages exceeding 500 kb [50]. Overall, tailed phages with dsDNA account for roughly 93% of all known phages, with *Siphoviridae* representing the most prevalent family [51].

### 2.2. Phage Life Cycles and Genome Packaging Mechanisms

The genome of complete phages encodes essential genes required for replication, structural integrity, and DNA packaging. Based on their replication strategies, phages can be classified into three main lifestyle types: lytic, temperate, and chronic. Lytic phages rapidly replicate within the host and induce cell lysis to release progeny [52]. Recent findings indicate that certain lytic phages may also establish persistent infection without immediate host lysis [53,54]. In contrast, temperate phages integrate into the bacterial chromosome or are maintained as extrachromosomal elements (e.g., plasmid-like maintenance in P1 phage [55]), remaining latent until triggered into a lytic cycle. Chronic phages, exemplified by M13 phage, replicate continuously and release progeny without lysing the host [56].

Phage packaging refers to the encapsidation of newly synthesized phage genomes into progeny particles. The terminase complex, composed of small (TerS) and large (TerL) subunits, recognizes and cleaves concatemeric phage DNA for packaging into preassembled capsids. The nature of the phage genome termini determines the packaging strategy [57]. Fixed termini include cohesive ends (cos site) found in λ and P2, and direct terminal repeats (DTR) found in T7 (short) and T5 (long). Alternatively, headful packaging generates a circularly permuted genome, as in T4, or genome ends derived from host DNA, as seen in Mu phage. Notably, certain phages possess proteins attached to their ends that facilitate their genome packaging, as in *Bacillus subtilis* phage 29 [58]. Traditionally, determining packaging mechanisms required in-vitro assays, but recent advances in bioinformatics have enabled reliable prediction from high-throughput sequencing data [59,60]. These insights have facilitated the development of phagemid systems—plasmids incorporating phage packaging signals—that enable the delivery of heterologous genetic cargo via engineered phage particles [22,23,36,37,38,61] (Table 1).

### 2.3. Host Recognition and Engineering of Receptor-Binding Proteins

Host recognition is a critical determinant of phage infectivity and a key target in the functional engineering of phage host range and specificity. In tailed phages, this process is largely mediated by structural elements located at the distal end of the tail, where receptor-binding proteins (RBPs)—such as tail fibers (TFs) and tail spike proteins (TSPs)—initiate contact with the host cell surface receptor [62,63]. Bacterial surface receptors vary depending on the type of host cell. In Gram-negative bacteria, common receptors include lipopolysaccharide (LPS) and outer membrane proteins, whereas in Gram-positive bacteria, receptors typically consist of peptidoglycan and teichoic acids [64]. Other surface appendages, such as pili and flagella, may also serve as phage receptors. In non-tailed filamentous phages such as M13, infection is initiated by the interaction between the virion minor coat protein P3 and the F-pilus of the host. Notably, protein P3 is widely exploited in M13 phage display technologies [65,66,67].

Among Gram-negative bacteria, LPS serves as a frequent target and consists of three main components: the lipid anchor (lipid A), a core polysaccharide, and a highly variable O-antigen [64]. Based on the presence or absence of the O-antigen, LPS is classified as either smooth or rough [68]. Phages that recognize the conserved core region tend to exhibit broader host ranges, whereas those targeting the variable O-antigen often display a narrow host range [63]. For example, the long tail fibers (LTF) of *E. coli* phage T4 bind both the LPS core and the outer membrane protein OmpC [69]. In *E. coli* phage λ, the tail tip protein gpJ binds the maltose porin LamB, while the side tail fiber (STF) protein interacts with OmpC [70]. Phage T7 binds rough LPS—lacking the O-antigen—through its tail fiber protein gp17 [71]. The phage PhiV10 recognizes the O157:H7 O-antigen of Shiga toxin-producing *E. coli* (STEC) (O157) via its tail spike proteins (TSPs), and phage EP75 employs four TSPs to detect various O-antigens from both *E. coli* and *Salmonella* [72,73]. Similarly, *Salmonella* phage S16, considered to have a broad host range, recognizes OmpC via its LTF gp37, while P22 phage detects the O-antigen via its TSPs [74,75].

Engineering of RBPs has enabled phage host range reprogramming (Table 2). For instance, the host range of *E. coli* phage P2 was altered to target *Shigella flexneri* M90T, *E. coli* O157:H7, and *Salmonella* by constructing hybrid tial fibers incorporating the C-terminal region of P1(S’), PhiV10, and S16, respectively [24,35]. Similarly, the λ phage STF protein was modified to enable the phage binding to the LPS O157 O-antigen [23]. In another example, the T3 phage tail fiber gp17 was partially replaced with that from *Yersinia* phage R, thereby enabling infection of *Y. pestis* strains IP2666 and YPIII. Moreover, the entire tail apparatus of phage T7 (gp11, gp12, and gp17) was replaced with that of *Klebisella* phage K11 to enable cross-genus targeting [76].

Phage RBPs have also been leveraged for functional purposes beyond infection. Engineered phages expressing reporter genes such as *lux* or HiBiT tags have been developed for the rapid detection of specific pathogens [30,31]. Additionally, RBPs have been conjugated to antimicrobial agents like pyocin and nisin, facilitating targeted delivery of these bactericidal molecules to susceptible bacterial strains [42,77]. These advances underscore the versatility of RBPs not only in modulating host range but also in enabling synthetic and therapeutic innovations in phage-based platform technologies (Table 2).

**Table 2:** Engineered Phages with Modified Tail Fibers for Host Range Expansion and Therapeutic Applications.

Phage Name	Phage Type	Original RBPs/Host	New RBPs/Host	Introduced Modification	Purpose and Application	References
λ	Temperate	LamB, OmpC	OmpC and LPS	P2-STF	Genetic engineering of bacteria in the mouse gut/Transduction capsid/dCas9 base editor	[22]
λ	Temperate	LamB, OmpC	OmpC and O-antigen (O157)	STF tail fiber that recognize O-antigen (O157)	Eliminate STEC/Transduction capsid/Cas12a	[23]
P2	Temperate	LPS	*Shigilla flexneri* M90T, *Escherichia coli* O-antigen O157	Hybrid long tail fiber genes (gpHG or gpH only) P1-S’ and P1-U’, or PhiV10 tail spike protein, respectively	Eliminate STEC/Transduction capsid/Cas9	[35]
P2_vir1_	Lytic	LPS	*Salmonella* (OmpC)	P2-gpH and S16-gp37	Expand the Host range	[24]
T7	Lytic	Rough LPS	-	Selective mutations in HRDRs of *gp*17	Expand host range/Transduction capsid	[61]
T7	Lytic	Rough LPS	*Klebsiella*	Swapping gp11, gp12, and gp17 with those of phage K11	Expand host range	[76]
T3	Lytic	-	*Escherichia coli* (BW25113)	Swapping T3-gp17 with T7-gp17	Expand host range	[78]
α15	Lytic	LPS	Tsx	Knock-In of gp38 from phage α17	Expand host range/Cas delivery	[25]
Others	R-type pyocin	*Pseudomonas*	*Escherichia coli* O-antigen (O157)	Utilize the C-terminal of the phiV10 tail spike protein	Expand host range/Bacteria killing agent	[77]
Others	Nisin-nanoparticles	-	MRSA	RBPs of Staphylococcal phage Sb-1	Expand host range/Bacteria killing agent	[42]

Abbreviations and Notes: RBP: receptor binding protein. HRDR: Host Range-Determining Region. STEC: Shiga toxin-producing *Escherichia coli*. gp17: Tail fiber protein of phage T7. STF: Phage l side tail fiber protein. gpH: P2 phage long tail fiber protein. gpH P1-S’: Tail fiber variant of phage P1. P1-U’: Phage P1 tail chaperone protein of S’. gp37: Long tail fiber protein of Salmonella phage S16. Tsx: Nucleoside-specific outer membrane protein. gp38: adhesin proteins of T-even phages in this case Phage α17 phage. MRSA: Methicillin-Resistant *Staphylococcus aureus*.

## 3. Engineering Strategies for Bacteriophages

Phage bioengineering has advanced beyond traditional mutagenesis techniques—such as ultraviolet light exposure or screening modified progeny after mixed infection (phage crossing) [11,79]—which were indirect and labor-intensive. Modern strategies now enable precise, targeted modifications based on phage properties, such as lifestyle (temperate or lytic), genome size, and host type. Temperate phages are generally easier to manipulate genetically, as their genomes can be modified similarly to chromosomal genes in bacteria. Reporter genes or selection markers can be inserted to facilitate screening [44]. While temperate phages are less favored for direct therapeutic applications, they are often utilized in constructing CRISPR-Cas-loaded capsid systems [22,23,35,36,37,38,44]. In contrast, lytic phages present greater challenges due to their rapid replication cycles, lower recombination efficiency, and the lack of a stable lysogenic phase. Thus, they require more robust engineering tools and effective selection methods. Lytic phages may be engineered in-vivo using recombineering systems and/or CRISPR-Cas tools or assembled in-vitro through synthetic methods. Screening of modified progeny can be accomplished by PCR, phenotypic selection [33], or insertion of a reporter gene [80].

A critical step after any genome manipulation is the successful recovery of infectious phage particles, known as “phage rebooting.” This process is highly sensitive to the engineering method used and the host cell selected for reactivation. 

### 3.1. Genetic Engineering Approaches

**Figure 2:**
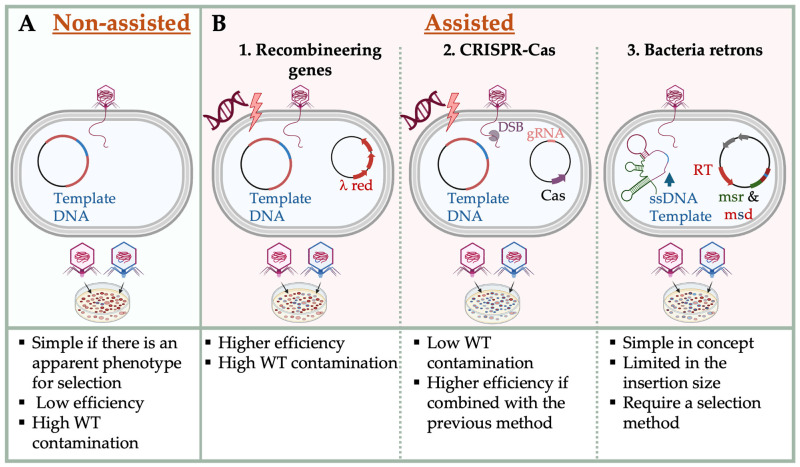
In-vivo Methods for Phage Engineering with Representative Mechanisms. (**A**) Non-assisted phage engineering through spontaneous homologous recombination between phage and donor DNA without auxiliary genetic tools. (**B**) Assisted phage engineering strategies, classified by the supporting system employed: (**B1**) the λ red recombineering system, utilizing Exo, Beta, and Gam proteins to facilitate homologous recombination with linear or circular donor DNA (ssDNA or dsDNA). (**B2**) CRISPR-Cas-assisted engineering system, where targeted double-stranded DNA breaks (DSBs) enhance recombination efficiency and eliminate wild-type (WT) phage via counterselection. (**B3**) Retron-based engineering, where bacterial retrons generate donor ssDNA through reverse transcription of ncRNA comprising msr and msd regions. In all panels, donor DNA is depicted in blue, homologous regions (HRs) in red, Cas nucleases in purple, and guide RNAs in light red. Phage genetic material may be introduced as infectious particles or via electroporation. These platforms enable precise genome editing in both temperate and lytic phages.

Phage in-vivo modification refers to the process of engineering phage genomes within a host cell, whether it is the native host or an intermediate host. Donor DNA may be introduced as circular or linear double-stranded (dsDNA) or single-stranded (ssDNA). Recombination between a phage genome and a donor plasmid carrying homologous regions (HRs) can occur spontaneously [25,33], but this process is significantly enhanced by host strains equipped with phage-encoded recombineering genes [81] or retroelements such as bacterial retrons [82]. Donor DNA may be introduced before phage infection or co-transformation along with the phage genome into the recombining host [83]. The optimal strategy depends on the host, DNA type, and engineering goal (Figure 2).

#### 3.1.1. Phage Recombineering System

Phage recombineering systems are specialized homologous recombination tools encoded by phages that allow for a precise genetic modification of bacterial and phage genomes. These systems typically function by expressing phage-derived exonucleases and annealing proteins that promote the integration of donor DNA via short homologous arms. The recombineering machinery has been characterized in several bacterial hosts and their phages, enabling diverse applications ranging from targeted mutagenesis to large-scale genomic rearrangements. In this section, we describe representative recombineering systems identified in *Escherichia coli*, *Mycobacterium*, and *Pseudomonas*, each offering unique capabilities for engineering both temperate and lytic phages.

**(a) *Escherichia coli*:** The λ red recombineering system is one of the earliest and best-characterized phage-encoded systems in *E. coli*. It was initially identified due to its recombination capability in a RecA-deficient strain [84,85]. This system consists of three phage-encoded proteins—Exo, Beta, and Gam—that complement each other’s functions [86,87]. Exo is a 5′-3′ exonuclease that degrades one strand of the dsDNA, generating ssDNA [88]. Beta is a recombinase that binds the single-stranded DNA tail and promotes pairing with its complementary sequence [89]. Gam inhibits degradation of dsDNA by the host RecBCD exonuclease complex [90]. In ssDNA applications, only Beta and Gam are required. This system has been widely used to insert, delete, or replace genes in *E. coli*, and related bacterial species [91,92,93], as well as in temperate *E. coli* phages [94,95] and lytic ones [96]. For efficient activity, the system requires 35–40 bp of homologous sequence flanking the donor DNA [97]. In *E. coli*, it is used for modifying temperate phages integrated on the chromosome [22,23,35] or as plasmids [44] and lytic phages, often in combination with CRISPR-Cas systems [98]. This system has also been adopted for use in other bacterial species such as *Yersinia* [99] and *Pseudomonas* [100].

In addition to the λ Red system, *E. coli* also harbors other phage-derived recombineering systems, such as those from the Rac prophage, known collectively as the RecE/T system. This system requires two proteins: RecE, which functions similarly to λ Exo, and RecT, analogous to λ Beta [101]. The RecE/T system has been successfully used in various genetic engineering contexts [102]. Similar homologous systems have been identified in other bacteria as well, including *Mycobacterium* [83,103,104] and *Pseudomonas* [105,106].

**(b) *Mycobacterium*:** Prior to the identification of phage-encoded recombination genes in *Mycobacterium*, genetic engineering of mycobacteriophages was achieved by constructing phage–plasmid hybrid systems and manipulating them in an intermediate host, typically *E. coli* [107,108,109]. However, advances in whole-genome sequencing led to the discovery of a RecE/T-like recombineering system in *Mycobacterium* phage Che9c [103]. This system comprises two phage genes: Gene 60, which encodes an exonuclease, and gene 61, which encodes a recombinase. Both genes are required for dsDNA recombineering, while only the recombinase is necessary when using ssDNA templates [104]. The breakthrough enabled the development of more efficient genetic engineering methods in *Mycobacterium*, most notably the bacteriophage recombineering with electroporated DNA (BRED) technique [83]. BRED involves the co-transformation of phage genomic DNA and a linear donor template DNA into recombineering-competent *Mycobacterium* cells. The donor DNA carries homologous arms of approximately 100 bp flanking the target gene. Recombinant plaques are then screened by PCR, resulting in scarless genetic modifications of the phage genome [83]. This method was initially applied to *M. smegmatis* and was later enhanced by integrating CRISPR-Cas9 as a counter-selection tool to improve screening efficiency. The combined system is referred to as CRISPY-BRED, or CRISPY-BRIP for bacteriophage recombineering with infectious phage particles [110].

**(c) *Pseudomonas*:** Similar to other bacterial species, *Pseudomonas* has been found to harbor recombineering systems encoded either by native elements or associated phages. These systems, collectively referred to as *Pseudomonas* Encoded Homologues Recombineering (PEHR) genes, include RecE/T-like modules and Red-like operons that facilitate homologous recombination [105,106]. One notable system is the RecTE_Psy recombineering pair identified in *P. syringae*, which is analogous to the *E. coli* Rac prophage RecE/T system. Additionally, a Red-like operon—termed the BAS system—was identified in *P. aeruginosa* phage Ab31 [106,111]. Both systems have demonstrated efficient dsDNA-mediated recombination activity in their respective *Pseudomonas* hosts [106,111,112]. To further enhance editing specificity and efficiency, PEHP systems have been integrated with CRISPR-Cas technologies. Notably, the CRISPR-Cas3 system (Class I, Type I-E), which targets DNA in a processive manner, was coupled with PEHR to successfully engineer *P. aeruginosa*, achieving high-efficiency phage genome modifications [113]. This combinatorial approach has opened the door to broader applications, including the deletion of large genomic regions, the insertion of functional genes, and the construction of customized lytic phages. In addition, the λ Red system from *E. coli* has been adopted to function in *Pseudomonas aeruginosa*, with some host-specific modifications. It was successfully expressed in *P. aeruginosa* to support recombination-based editing [100,114]. When combined with Cas9 or Cas12a effectors, this hybrid system has proven effective for editing *Pseudomonas* phage genomes and producing recombinant phages with altered properties [115].

In addition to the mentioned examples, other recombineering systems, such as the flippase system (FLP), a site-directed recombination system discovered in yeast, is also used for phage engineering [116]. Beyond genome editing, all these systems are increasingly being integrated into phage-based platforms for therapeutic development.

#### 3.1.2. CRISPR-Cas-Assisted Phage Editing

Clustered Regularly Interspaced Short Palindromic Repeats (CRISPR) and their associated proteins (Cas) originally functioned as a bacterial and archaeal adaptive immune system to defend against invading genetic material, including DNA or RNA from phages and other mobile genetic elements. CRISPR systems are composed of an array of short repeat sequences interspersed with unique spacer sequences derived from foreign elements, transcribed into CRISPR RNAs (crRNAs). These crRNA guide Cas nucleases to complementary target sequences, where site-specific cleavage is induced. Due to their modularity, programmability, and precision, CRISPR-Cas systems have emerged as powerful tools for genome editing across diverse domains—including eukaryotic, prokaryotic, and viral systems [117,118].

In phage engineering, CRISPR-Cas systems are widely utilized not only to facilitate precise genomic modifications but also as a counter-selection mechanism to eliminate wild-type (WT) phage contamination. Class II CRISPR systems, which consist of a single-effector protein such as Cas9, Cas12a, or Cas13a, are particularly well-suited for phage editing due to their simplicity and efficiency. These systems are used either independently [80,119] or in combination with recombination genes [98,115,120,121].

**(a) Cas9 (Type II):** Cas9 is a widely utilized RNA-guided endonuclease that introduces double-stranded breaks (DSBs) at specific locations within the DNA. These breaks are directed by either a crRNA/tracrRNA complex or a single-guide RNA (sgRNA) that complements the target sequence. The most commonly used variant in phage genome engineering is *Streptococcus pyogenes* Cas9 (SpCas9), typically delivered via the plasmid pCas9. Efficient target recognition and cleavage by Cas9 require the presence of a short conserved sequence downstream (3′ end) of the target site known as the protospacer adjacent motif (PAM), which in the case of SpCas9 is generally NGG, where N is any nucleotide G, C, A or T, [122]. Several engineered Cas9 variants with relaxed PAM requirements have been developed, including those recognizing NNG (less strict) [123], NRN or NYN (semi-PAMless), where R is A or G and Y is T or C [124,125], and even NNN (fully PAMless) motifs [126].

Cas9-assisted phage engineering systems typically consist of three components: A plasmid encoding the Cas9 protein, a guide RNA (gRNA) specific to the phage genome target, and a donor DNA template carrying the intended genetic modification. The donor template enables homologous recombination to repair the DSB introduced by Cas9, incorporating the desired edit in the process [119,127]. Both single and dual gRNAs have been used to flank the targeted region, enabling precise deletions, insertions, or sequence replacements [80,127,128]. This method has been successfully applied to modify large virulent phages, including *E. coli* (T4), *Klebsiella pneumoniae* phiKpS2, and *Staphylococcus aureus* K phage [127,128,129]. The design and pre-screening of guide RNAs are crucial to ensure high specificity and minimize off-target cleavage [80,129]. Importantly, the DSB introduced by Cas9 serves as a counter-selection mechanism, effectively eliminating wild-type (unmodified) phage genomes that lack the intended sequence change. To avoid cleavage of the successfully modified phage, the donor DNA is engineered to disrupt the original PAM site or guide RNA recognition sequence [80,98].

Additionally, the efficiency of plating (EOP) can be measured prior to donor DNA introduction to evaluate the cutting efficiency of each gRNA—an essential step for optimizing editing outcomes [98]. Combining Cas9-mediated cleavage with the recombineering system has enabled efficient construction of deletion and insertion mutants in phages infecting *E. coli* (T3, T7, and T5) [98] and *P. aeruginosa* [121], as well as *Mycobacterium* [110].

**(b) Cas12a (Cpf1, Type V):** Cas12a, formerly known as Cpf1, is a single-effector nuclease belonging to the Class II, Type V CRISPR-Cas system. Similar to Cas9, Cas12a targets DNA, and its effector is a single protein. However, compared to Cas9, Cas12a mediates targeted genome editing by inducing site-specific double-stranded DNA breaks (DSBs); however, it differs from Cas9 in several important respects that make it particularly attractive for phage engineering. First, Cas12a recognizes a T-rich PAM sequence (typically TTTV, where V = A/C/G), which expands the range of accessible genomic targets, particularly in AT-rich regions where Cas9 may be less effective [130]. Second, instead of generating blunt-end breaks like Cas9, Cas12a introduces staggered cuts with 5-nucleotide 5′ overhangs, which may facilitate more efficient recombination and ligation during DNA repair processes. This structural feature can be advantageous for precise insertions or directional cloning.

Cas12a is also capable of processing its own CRISPR array without the need for a separate trans-activating crRNA (tracrRNA), allowing for simplified guide RNA architecture. This feature facilitates multiplex targeting and reduces the genetic burden of the editing system, particularly in compact constructs utilized in phage engineering. Recent studies have demonstrated the utility of Cas12a in modifying phage genomes. For example, Cas12a has been used alone to engineer *E. coli* phage T4, where the introduction of edits was achieved without the assistance of additional recombineering genes [131]. Similarly, Cas12a has been employed to edit cyanophages—viruses infecting cyanobacteria—which are emerging as models for aquatic microbial ecology and synthetic biology [132]. In addition to solo applications, Cas12a has also been successfully combined with homologous recombination systems, such as the λ Red operon, to enhance editing efficiency and specificity in phages infecting *Pseudomonas* phage engineering [115]. This combination has allowed for precise insertions, deletions, and base substitutions in large lytic phage genomes.

The distinct mechanistic features of Cas12a, along with its compact size, self-processing crRNA capability, and staggered DSB profile, make it a highly flexible and effective tool for phage genome engineering, particularly when PAM site constraints or off-target risks pose challenges for Cas9-based approaches.

**(c) Cas13a (Type VI):** Cas13a is the signature effector protein of the Class II, Type VI CRISPR-Cas system and is fundamentally distinct from DNA-targeting nucleases such as Cas9 and Cas12a. Unlike these systems, Cas13a specifically targets single-stranded RNA (ssRNA), enabling programmable degradation of transcripts without modifying the genome [133,134]. This makes it ideal for transcript-level regulation, gene knockdown, or selective elimination of wild-type phage through RNA interference. Importantly, Cas13a does not require a protospacer adjacent motif (PAM), affording flexible target selection across diverse phage and bacterial RNA sequences [135].

In phage genome engineering, Cas13a has been utilized primarily as a post-editing selection tool. A representative two-step enrichment strategy was demonstrated by Adler et al. [136], who engineered *E. coli* phage T4. Initially, phages were edited through homologous recombination using a donor plasmid. In the second step, progeny were propagated in *E. coli* expressing Cas13a from *Leptotrichia buccalis* (LbuCas13a) along with a guide RNA targeting an essential wild-type phage transcript. This setup enabled efficient elimination of unedited phages, resulting in 100% enrichment of the desired mutant. This system has also been extended to jumbo phages. In a notable example, Guan et al. [137] edited *Pseudomonas aeruginosa* jumbo phage KZ by coupling LbuCas13a with the anti-CRISPR protein AcrVIA1, which suppresses Cas13a’s collateral RNase activity during the editing phase—thereby minimizing host toxicity and enabling efficient propagation of the engineered progeny.

Beyond genome editing, Cas13a can be exploited for transcriptome reprogramming, such as silencing bacterial virulence genes or selectively targeting phage transcripts involved in replication. Its collateral RNase activity, while potentially cytotoxic, can also be harnessed for broad-spectrum antimicrobial action if appropriately controlled. In summary, Cas13a broadens the utility of CRISPR-based phage engineering into the RNA realm. Its PAM-independence, transcript-level specificity, and compatibility with anti-CRISPR regulation make it a valuable complement to Cas9 and Cas12a for sophisticated phage manipulation and WT phage counter-selection.

#### 3.1.3. Retron-Mediated Genome Modification

Retrons are bacterial retroelements originally discovered in the early 1980s as DNA-RNA hybrid molecules composed of multi-copy, single-stranded DNAs (msDNAs) produced by the soil bacterium *Myxococcus xanthus* [138]. These msDNAs consist of a covalently linked hybrid structure in which a single-stranded RNA (msr) is attached to a segment of RNA (msr). The msr–msd duplex is partially reverse transcribed by a retron-encoded reverse transcriptase (RT), which is associated with a non-coding RNA (ncRNA) [139]. The msd region serves as the DNA template, while the msr region functions as a primer during reverse transcription, generating RT-DNA in-vivo [140,141,142]. Retron-like systems have since been identified across a range of bacterial species [143], and their natural function has recently been linked to bacterial defense. Specifically, the retrons are known to sense inhibition of the host cell RecBCD complex—an essential component of DNA repair—and to activate abortive infection responses via an associated effector protein, thereby protecting bacteria from phage propagation [144].

In synthetic biology, retrons have been repurposed as tools for precise genome editing through the generation of intracellular single-stranded donor DNA (RT-DNA). In this application, the retron ncRNA and RT cassette are used to produce a donor template that integrates into the genome via homologous recombination, often in conjunction with recombineering systems such as single-strand annealing protein (SSAP) and host-derived single-stranded DNA-binding proteins (SSBs). This retron-derived RT-DNA strategy has enabled scarless and multiplex genome editing in bacteria, yeast, and mammalian cells [145]. Recent improvements in retron design—such as engineering the ncRNA to enhance RT-DNA yield—have allowed for the introduction of precise single-nucleotide variants, short insertions or replacements (up to 8 bp), and deletions of up to 100 bp across multiple loci simultaneously in a prokaryotic system. This has been demonstrated in *E. coli* and extended to eukaryotic cells such as yeast and human cells HEK293T with the assistance of Cas9 to facilitate recombination and reduce off-target effects [146]. However, the advantage of Cas9 co-expression may vary depending on the host species and editing context. In certain bacterial systems, efficient retron-based editing has also been achieved without the use of CRISPR-Cas, indicating that further experimental validation is needed to standardize this approach.

Importantly, retron technology has also been adapted for phage genome editing. For example, RT-DNA produced by engineered retrons has been used to introduce single-base substitutions, small deletions, and targeted insertions into phage genomes [82]. The efficiency of retron-based editing depends on phage species, genome structure, and host recombineering capacity. Overall, retron-mediated genome editing represents a powerful and flexible platform for precise phage and bacterial genome engineering. It complements other editing tools such as recombineering and CRISPR-Cas systems and expands the synthetic biology toolkit by enabling high-throughput, template-guided modifications without the need for in-vitro DNA preparation or plasmid delivery.

### 3.2. In-Vitro Synthetic Engineering Platforms

**Figure 3:**
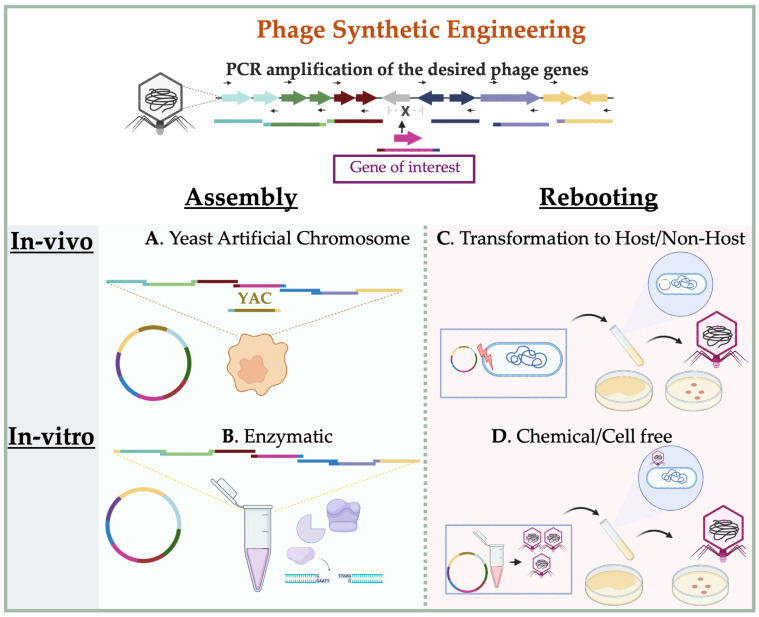
Overview of Synthetic Phage Engineering Strategies: Assembly and Rebooting Options. This figure shows the two main steps in synthetic phage engineering: genome assembly and phage rebooting. (**A**) YAC-based assembly performed in yeast cells (in-vivo). (**B**) Gibson assembly performed in-vitro using DNA fragments with overlapping ends. (**C**) In-vivo rebooting by transforming assembled genomes into native hosts, intermediate strains, or L-form bacteria. (**D**) In-vitro rebooting using TXTL (cell-free transcription–translation) systems. Steps (**A**,**C**) are in-vivo methods; (**B**,**D**) are in-vitro methods. Resulting phages can be further propagated in suitable bacterial hosts.

Phage synthetic engineering encompasses two primary steps: The assembly of phage genome components and the subsequent reactivation of these components into a functional state. This methodology does not require a selection step, as phage fragments can be generated directly through PCR amplification or chemical synthesis [78]. These fragments are then assembled either in-vivo using yeast cells [147] or a genetically engineered bacterial host, referred to as a “stepping stone host” [27]. In-vitro assembly can also be achieved enzymatically using various chemical or molecular methods, such as Gibson assembly [148], Golden Gate assembly [149], or a system involving exonuclease III (Exo III) and a phage packaging signal, such as in the iPac method [150]. Each method has its own advantages, requirements, and limitations, and the optimal choice depends on the characteristics of the phage and the compatibility of the host used for propagation. The rebooting step, which follows genome assembly, can be achieved either in-vivo (e.g., by transformation into a suitable bacterial host) or ex vivo using a cell-free transcription–translation (TXTL) system [151,152,153]. These approaches facilitate the generation of infectious phage particles from synthetic DNA and bypass host-related barriers. A simple illustration of synthetic phage engineering methods is shown in Figure 3.

#### 3.2.1. Synthetic Assembly of Phage Genomes

Synthetic assembly of phage genomes involves the in-vitro reconstruction of complete viral genomes from PCR-amplified or chemically synthesized DNA fragments. This approach enables modular manipulation of phage genomes without relying on intracellular recombination, providing greater flexibility for engineering large or complex constructs. Multiple strategies have been developed to facilitate this assembly, including yeast-mediated recombination and various enzyme-based systems. The following sections outline representative methods, each with unique advantages tailored to different phage types and engineering goals (Figure 3).

**(a) Yeast-Based (YAC) Systems**: The Yeast Artificial Chromosome (YAC) system is a method optimized for cloning and assembling large DNA fragments—up to several hundred kilobases—using *Saccharomyces cerevisiae* as the host. In this system, DNA fragments are introduced into yeast spheroplasts (cells with enzymatically removed walls), where in-vivo homologous recombination and gap repair mechanisms facilitate the assembly of complete constructs [154]. YAC vectors typically incorporate essential yeast chromosome components: a centromere (CEN), two telomeres (TEL), and an autonomously replicating sequence (ARS), as well as a selection marker. These components, approximately 2.4 kb in size, ensure stability, segregation, and replication of the resulting recombinant plasmids in yeast [155]. Historically, YAC systems have been used in large-scale genome mapping and manipulation for both prokaryotic and eukaryotic organisms [156,157].

In the context of synthetic phage engineering, YAC systems allow the reconstruction of phage genomes from PCR-amplified fragments carrying homologous ends to YAC vector elements as shown in Figure 3A. Correct positioning of the YAC components within the phage sequence is critical and requires prior knowledge of the phage’s DNA packaging strategy [158,159,160]. Once assembled in yeast, the YAC-phage constructs can be extracted and rebooted either by transformation into the original bacterial host, an intermediate host [76] or via chemical rebooting protocols [159]. For Gram-positive bacteria such as *Staphylococcus aureus*, which are generally less amenable to transformation, the assembled YAC constructs are often first propagated in *E. coli* to increase plasmid yield. Subsequently, they are transferred to L-form cells, which are cell wall-deficient variants that allow uptake of large DNA constructs and enable phage rebooting [161]. This method has proven effective for assembling and activating synthetic genomes of phages infecting *E. coli*, *Klebsiella*, *Salmonella*, and *Pseudomonas*, typically with genome sizes ranging from 37 to 44 kb and has also been successfully applied to engineer functional modifications, such as tail fiber substitutions in T3 and T7 [76], assembly of a 66 kb *P. aeruginosa* JG024 [158], *S. aureus* phage SA75 [161], and deletion of 10 hypothetical genes in *P. aeruginosa* phage S4 [160]. Nevertheless, efficiency can vary depending on factors such as host strain, genome packaging compatibility, and innate bacterial antiviral defense systems [158] or the rebooting method [159]. Therefore, optimization of both assembly and rebooting conditions remains essential for reliable recovery of viable phages from YAC constructs.

**(b) Gibson assembly, Golden Gate, and iPac:** Gibson assembly is a widely used and versatile cloning technique that enables the seamless joining of multiple DNA fragments with overlapping ends in a single-tube isothermal reaction Figure 3B. The assembly relies on the coordinated action of 5′ exonuclease, DNA polymerase, and DNA ligase, which together digest, anneal, fill in gaps, and seal nicks between adjacent DNA fragments [148]. DNA fragments can be prepared using PCR or restriction digestion and are typically designed to include ~40 bp of overlapping sequence for optimal efficiency [162]. The effectiveness of Gibson assembly in phage genome construction depends on factors such as genome size, number of fragments, and sequence complexity. For example, Ando and colleagues successfully applied this method to assemble wild-type phage genomes, including *E. coli* phages (T3, T7 and λ), *Salmonella* phages (SP6 and P22), *Pseudomonas* phage gh-1, and Mycobacteriophages (B1, D29, and TM4) [78]. In their approach, each phage genome was reconstructed from 4 to 5 fragments—either PCR-amplified or chemically synthesized—with 28 to 65 homologous arms. Both linear and circular forms of the assembled genomes were used, with genome sizes ranging from 38 to 52 kb. This method also enabled the engineering of functional mutants, such as deletion of the C2 repressor gene in *Salmonella* phage P22 to produce a constitutively lytic mutant (P22Dc2), substitution of T3 tail fiber with that of T7 to expand host range, and insertion of reporter genes (e.g., Nanoluc into D29 and *LacZ* into T7) in place of non-essential regions. Rebooting of these phages was achieved via transformation into the native host, an intermediate host, or using a TXTL cell-free system (Figure 3C,D and Table 3) [78]. In direct comparisons, Gibson-assembled T7 phages showed higher rebooting efficiency than those assembled using YAC vectors, both in-vivo and in-vitro. This improved performance may be attributed to the absence of yeast-derived contaminants or processing artifacts [159]. As the size and complexity of synthetic phage constructs increase, the choice and optimization of the rebooting method become even more critical and are discussed in Section 3.2.2.

Golden Gate assembly is another modular cloning strategy that uses the activity of the type IIS restriction enzyme and T4 DNA ligase to concatenate multiple DNA fragments in a one-pot reaction [149]. Unlike conventional restriction enzymes, type IIS enzymes cleave outside of their recognition sites, generating unique 4–5 nucleotide overhangs that guide the directional ligation of fragments. Although this method is typically limited to assemblies involving fewer than 10 fragments, recent improvements in fragment design, junction selection, and ligation conditions enabled the successful construction of a 40 kb T7 phage genome from 52~800 bp fragments [166]. Another emerging strategy is the in-vitro packaging-assisted DNA assembly (iPac), which couples the activities of exonuclease III with the packaging mechanism of λ phage. In this method, PCR-amplified DNA fragments bearing ~50 bp homologous overlaps are assembled and subsequently packaged into λ capsids using the λ packaging signal, thereby bypassing the need for bacterial transformation [150]. In addition to λ phage itself, this approach has been extended to other *E. coli* phages, including T1, T3, T7, and phi80, with total genome sizes ranging from 38 to 49 kb. The iPac method provides an alternative and useful approach to engineer phages of transformation-resistant hosts [150]. Together, these enzymatic assembly strategies provide complementary tools for phage genome reconstruction, each offering distinct advantages in terms of scalability, sequence flexibility, and downstream compatibility with rebooting systems.

#### 3.2.2. Rebooting Engineered Phage

The final and most critical step in synthetic phage engineering is to restore the assembled genome to a biologically functional, infective form commonly referred to as rebooting. The success of this process depends not only on the phage genome itself but also on the characteristics of the rebooting host and the assembly method used. Selecting an appropriate rebooting system is essential to recover viable, genetically modified phage particles. Currently, two major approaches are available: In-vivo rebooting via transformation into live bacterial hosts, and in-vitro rebooting using cell-free transcription–translation systems.

Recent large-scale studies have evaluated the factors influencing rebooting efficiency. For instance, Cheng et al. [27] systematically assessed 126 phages spanning a range of genome sizes (20.7 to 299.5 kb) and phylogenetic lineages (*Myoviridae*, *Siphoviridae*, *Podoviridae*, *Ackermannviridae*, and unassigned phages), infecting diverse hosts including *E. coli*, *S. enterica*, *K. pneumoniae*, *P. aeruginosa*, and *A. baumanii.* Using a custom-engineered *E. coli* DH10B strain equipped with 10 rare and common tRNAs, they successfully rebooted 90 out of 126 phages, whose genome sizes ranged from 20.7 to 156 kb. Their results showed that smaller genome size and the presence of phage-encoded DNA polymerase positively correlated with in-vivo rebooting efficiency. On the other hand, in-vitro cell-free rebooting outcomes were shown to be more sensitive to the assembly method, particularly with regard to fragment purity and reaction byproducts [159]. A summary of the methodologies used to engineer some of the examples discussed in this review is provided in Table 3.

**(a) In-vivo rebooting in native and Non-native hosts, including L-form:** In-vivo rebooting involves restoring the infectivity of a synthetic phage genome by introducing it into a live bacterial host cell. While using the native host is generally preferred to maintain phage–host specificity and ensure efficient propagation, engineered intermediate hosts are often employed to overcome transformation barriers, improve rebooting yield, or support phages infecting genetically intractable bacteria. For example, *E. coli* strain 10G has been successfully used for rebooting Gibson assembled genome of *Pseudomonas* phage gh-1 (37.7 kb) and *Salmonella* phage SP6 (43.7 kb) [78]. Similarly, the *E. coli* strain DH10B, modified to express both common and rare tRNAs and λ red recombination functions, has enabled the efficient rebooting of cross-genus (*Salmonella* and *Klebsiella*) and cross-order (*Pseudomonas* and *Acinetobacter*) phages [27]. In case the target bacterium is Gram-positive and poorly transformable, rebooting can be achieved using L-form bacteria—cell wall-deficient variants that facilitate the uptake of large DNA molecules. For instance, Listeria-derived L-form have been employed to reboot phages infecting listeria, *Bacillus*, and *Staphylococcus* species [167]. Specifically, L-forms of *S. aureus* RN4220, induced by treatment with ampicillin and lysozyme, were used to reboot large-genome phages such as *Stapyllococcus* phages SA75 (43 kb) and phage K (148 kb), as well as *Enterococcus* phages from genomic DNA, including YAC-assembled constructs [161].

**(b) In-vitro rebooting (TXTL Cell-Free system):** The cell-free transcription and translation (TXTL) system is a transformation-independent strategy for rebooting synthetic phage genomes and has emerged as a powerful tool in the field of synthetic biology. Originally developed to decipher the genetic code [168], TXTL systems now enable rapid, modular synthesis of functional biological entities in-vitro, bypassing the need for living host cells [169]. TXTL platforms are typically derived from bacterial lysates—most commonly from *Escherichia coli*—in which endogenous genomic DNA has been removed while retaining the essential transcriptional and translational machinery [152]. Among them, the modular *E. coli* TXTL (mTXTL) system is widely used for synthetic phage biology. Other bacterial systems have also been developed to support host-specific applications [170]. Using *E. coli* TXTL system, researchers have successfully rebooted a broad range of phages from either genomic DNA or synthetically assembled genome fragments. These include well-characterized phages such as *E. coli* phages T7 and T4, as well as *Salmonella* phages FelixO1 and S16, spanning a range of genome sizes from compact to large [78,153,159,164]. The efficiency of TXTL-mediated rebooting is influenced by the preceding genome assembly method. For example, T7 phage assembled using Gibson assembly produced higher phage titer than those generated via the YAC approach, a difference attributed to the carryover of inhibitory yeast components from YAC plasmids extractions [159].

In addition to its modularity and flexibility, TXTL offers two major advantages over in-vivo rebooting. First, the absence of living cells eliminates cytotoxic constraints, enabling the expression and assembly of phages that encode toxic or lytic genes without compromising system stability. Second, TXTL systems avoid the introduction of bacterial endotoxins such as lipopolysaccharides (LPS), which are commonly present in live-cell phage propagation. This characteristic significantly enhances biosafety and makes TXTL particularly suitable for the production of phages intended for therapeutic applications [171].

Despite these advantages, TXTL also has some limitations. The system can be cost-prohibitive for large-scale or high-throughput applications, and the rebooting efficiency may be lower for phages with large genomes or those that require complex host-specific modifications. Overall, the TXTL platform has expanded the phage engineering toolkit by enabling precise, cell-free recovery of infectious phage particles, thereby facilitating the development of next-generation engineered phage therapeutics.

## 4. Applications and Innovations in Engineered Phage Platforms

Recent advances in synthetic and functional phage engineering have enabled the development of versatile applications that extend beyond natural lytic or temperate phage biology. Engineered phages are now being explored as programmable antimicrobial agents, precision diagnostic tools, and targeted delivery platforms. This section highlights key innovations across four main application areas.

### 4.1. Host Range Expansion and Targeting

One of the earliest and most actively developed applications of engineered phages is the expansion or redirection of host specificity. By modifying receptor-binding proteins (RBPs)—including tail fibers and tail spike proteins—phages can be retargeted to infect previously resistant strains or even cross bacterial genera. This strategy enables the rational design of custom phages to target emerging or drug-resistant pathogens.

For example, the host range of *E. coli* phage P2 was broadened to target *Shigella flexneri*, *E. coli* O157:H7, and *Salmonella* via C-terminal tail fiber swapping using donor sequences from P1(S’), PhiV10, and S16, respectively [24,35]. Similarly, modification of the λ side tail fiber (STF) allowed recognition of *E. coli* O157 antigens [23], and T3 phage was reprogrammed to infect *Yersinia pestis* by partial replacement of its gp17 tail fiber protein. Complete tail module exchange has also enabled phages like T7 to infect *Klebsiella* species [76]. Nevertheless, host resistance to phage infection remains a critical limitation in clinical use. Engineered cocktails combining multiple receptor targets, or sequential phage applications, are being investigated to suppress resistance emergence and sustain bactericidal activity [172]. Nevertheless, other approaches, such as phage training methods, which depend on phage evolution pressure, are also used to expand the phage host range [173].

### 4.2. CRISPR-Cas Delivery via Phage Capsids

CRISPR-Cas-loaded phage capsids, often termed “Cas-capsids,” represent a transformative approach to programmable antibacterial therapy. These systems use phage particles to deliver CRISPR-Cas payloads that target specific bacterial genes, such as resistance determinants, enabling sequence-selective killing or gene modulation [22,23,35,36,37,38,44]. For instance, Brödel et al. [22] demonstrated the use of λ phage capsids loaded with dCas9 for targeted repression of β-lactamase genes in *E. coli* colonizing the mouse gut. In another example, engineered lytic phages of the T-even family were used to deliver active CRISPR-Cas constructs—termed CAPs—either alone or in conjunction with wild-type phages, showing therapeutic efficacy in murine models [25].

### 4.3. Biocontained Non-Replicative Phage Therapeutics

To enhance clinical safety, engineered phage platforms have incorporated biocontainment strategies to prevent unintended replication. Two distinct formats have emerged: (i) non-replicative Cas-capsids derived from temperate phages and (ii) split-genome systems for lytic phages.

**Figure 4:**
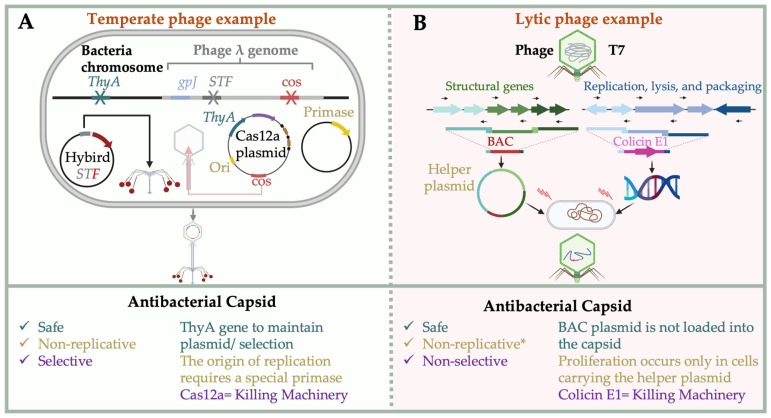
Recent Advancements in Antibacterial Phage Capsid Systems. (**A**) CRISPR-Cas12a-based antimicrobial capsid system using the temperate λ phage [24]. (**B**) Biocontained antibacterial capsid system using the lytic T7 phage to deliver colicin E1 [41]. In (**A**), a CRISPR-Cas12a plasmid lacking a primase and antibiotic resistance gene. The plasmid includes a phage origin of replication and a thymidylate synthase gene (*thyA*) for selection in a Δ*thyA* host, and it is packaged into λ phage capsids via the cos packaging signal. Tail-related genes are STF and gpJ, which encode the λ side tail fiber and tail tip proteins, respectively. In (**B**), a BAC-based helper plasmid expresses the T7 phage structural genes. The remaining T7 genome, including replication and packaging genes along with the colicin E1 expression cassette, is assembled separately and introduced into a bacterial strain carrying the helper BAC plasmid for particle production.

In Cas-capsid systems, plasmids encoding CRISPR-Cas systems, antibiotic markers, and replication origins are packaged into phage capsids using engineered temperate phage-derived packaging signals. To eliminate replicative potential and antibiotic resistance concerns, Galtier et al. and Brödel et al. developed a dual-plasmid system that separates the phage replication origin from the primase gene [22,23]. Galtier et al. used a thymidylate synthase gene (*thyA*) in place of antibiotic markers. Packaging occurs in a Δ*thyA* strain, producing safe and efficient antimicrobial agents [22,23] (Figure 4A).

For lytic phages, non-replicative variants have been constructed by splitting structural and replication components. Kiga et al. assembled the structural genes of phage T7 into a bacterial artificial chromosome (Virion-BAC), while the remaining genome—including replication, packaging, lysis, and the payload (e.g., colicin E1)—was introduced separately. These particles only replicate in cells containing the Virion-BAC helper plasmid, ensuring strict biocontainment (Figure 4B) [40]. Similar systems have been demonstrated using capsid or tail-deficient rebooting hosts [61,78]. These approaches aim to ensure strict biocontainment and minimize the risk of horizontal gene transfer.

In addition to biocontainment, host immune responses pose a significant barrier to phage-based therapies. Circulating phages are often rapidly cleared by innate immune mechanisms such as complement activation or neutralizing antibodies, limiting their bioavailability. Strategies such as PEGylation of capsids or transient immunosuppression have been explored to prolong circulation time and enhance therapeutic efficacy [174,175].

### 4.4. Diagnostic and Antimicrobial Payload Delivery

Engineered phages have also shown promise in diagnostics and precision delivery of antimicrobials. Diagnostic phages carrying reporter genes such as lux or HiBiT allow rapid detection of specific pathogens with high sensitivity [30,31]. By incorporating tags into the phage genome, these engineered systems facilitate quantifiable bioluminescent or luminescence-based detection.

Phage particles have also been repurposed as delivery vehicles for therapeutic proteins and antimicrobial peptides. RBPs fused to bacteriocins such as pyocin or nisin have been used to selectively deliver payloads to target bacteria [42,77]. CRISPR-based delivery of gene-editing systems, as well as programmable base editors, further expands the therapeutic utility of phage-derived platforms. Collectively, these innovations demonstrate how engineered phages can function as modular and programmable tools for targeted antimicrobial therapy, diagnostics, and delivery—paving the way for next-generation precision medicine.

## 5. Conclusions and Future Perspectives

In this review, we have presented a comprehensive overview of the current strategies in the synthetic and functional engineering of bacteriophages, with emphasis on practical applications that extend far beyond classical lytic therapy. We have examined a wide range of engineering approaches applicable to both temperate and lytic phages, including in-vivo genetic tools such as recombineering systems, CRISPR-Cas technologies, and retron-based editing platforms, as well as in-vitro synthetic methods involving modular genome assembly and cell-free rebooting. In-vivo-assisted systems, including phage-encoded recombineering genes and CRISPR-Cas elements, enable precise genomic modifications and targeted gene disruptions, significantly improving the success rate of engineering phage variants. Retrons further expand the toolkit by facilitating multiplexed and scarless genome edits. Meanwhile, in-vitro synthetic engineering platforms—such as YAC-based assembly, Gibson and Golden Gate cloning, and in-vitro packaging (iPac)—have made it possible to build and reboot phage genomes without reliance on native host biology, thereby circumventing species-specific transformation limitations.

The applications of these engineered phage platforms are broad and expanding. We highlighted host range redirection through RBP engineering, CRISPR-Cas delivery via capsid packaging (Cas-capsids), the construction of biocontained non-replicative phages for enhanced clinical safety, and the development of phages as diagnostic agents and delivery vehicles for antimicrobial proteins or gene editors. These innovations are setting the stage for precision antibacterial interventions, synthetic microbial control strategies, and new modalities in therapeutic delivery. However, despite promising in-vitro performance, the pharmacokinetics and pharmacodynamics (PK/PD) of engineered phages in-vivo remain poorly understood. Clearance rates, tissue penetration, and optimal dosing regimens are all active areas of investigation using animal models and imaging tools [176].

Future directions in this field include incorporating machine learning (ML) and Artificial Intelligence (AI), which can aid in high-throughput screening and predicting phage infectious strains for phage therapeutic applications. Such tools are crucial in experimental setups where wet experiment design is challenging and when time and manual labor are limited. The current surge in phage and bacterial genomic data has facilitated the development of these tools, allowing for the precise prediction of suitable phages using available host genomic data. Although actual effectiveness highly depends on various host-related factors [177]. Recently, these platforms and models have been trained with receptor-binding protein databases and host interaction profiles to select phages with optimized infectivity in silico, which are later validated with wet experiments [178,179]. It is also believed that in the near future, the integration of these models with the rapid advancement of synthetic biology will lead to a new era of phage therapy based on case-by-case analysis, achieved by what Pirnay described as a bedside energized anti-microbial unit [180]. Additionally, constructing minimal phage genomes—removing non-essential genes—may enhance stability, reduce immunogenicity, and simplify regulatory compliance (e.g., de novo synthetic assembly of minimal *Pseudomonas* phage genomes demonstrated in recent yeast TAR rebooting work [160].

**Figure 5:**
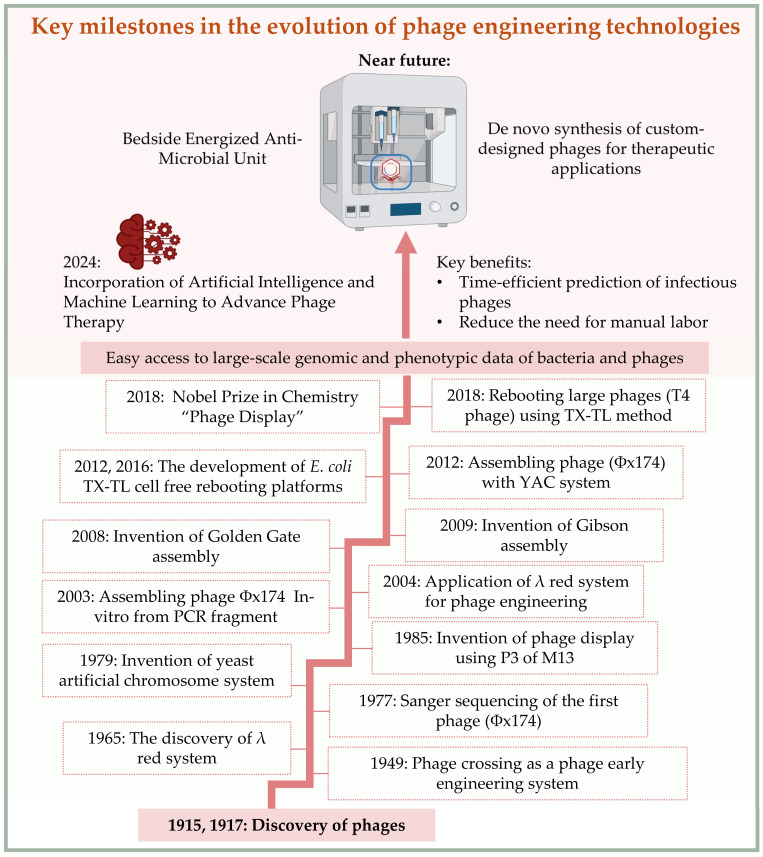
Chronological Milestones in The Development of Phage Engineering Systems from The Early 20th Century to Future Prospects. The lower section of the figure shows selected key discoveries and milestones that contributed to the current advancement of phage engineering, while the upper section describes the current and future expected advancement of synthetic phage engineering for therapeutic applications. The upper section begins with the discovery of phages [3,4] and it lists key achievements and innovations such as: the confirmation of phage crossing between closely related phages [11], the discovery of the λ red system [85], the complete sequencing of the first phage [15], the invention of phage display [190], and the Yeast Artificial Chromosome system (YAC) [154]. It also includes the first application of the λ red system in phage engineering [94], the innovations of Golden Gate assembly [149] and Gibson assembly [148], the assembling of phage Φx174 in-vitro [191], the assembling of Φx174 in-vivo using YAC system [147], and the development of *E. coli* TX-TL platforms for phage rebooting [152,192]. Additionally, it covers the rebooting of T4 phage using the TX-TL method and concludes with the global burst in whole-genome sequencing and the availability of large genomic data, which is leading to a new era in phage engineering. As machine learning and artificial intelligence are integrated into phage engineering systems, we anticipate reaching a new era of phage engineering platforms [180].

As the engineering landscape evolves, phages are set to become modular biotechnological tools for applications ranging from infection control and microbiome editing to programmable therapeutics [181,182,183,184,185,186]. This is enabled by the advancement of phage engineering systems, which have rapidly progressed over the years, as shown in Figure 5, now reflecting a convergence of synthetic biology, systems microbiology, and therapeutic innovation, offering unprecedented potential for safe, precise, and adaptable microbial interventions. To realize these clinical potentials, standardized protocols for large-scale phage production under Good Manufacturing Practice (GMP) conditions are essential. Regulatory frameworks for engineered phages are still evolving, and close coordination with agencies such as the FDA and EMA will be vital to translate synthetic phage products into approved therapeutics [187,188,189].

## Figures and Tables

**Table 1:** Representative Engineered Phages and Their Functional Applications Across Therapeutic, Diagnostic, and Delivery Platforms.

Function	Phage Name	Cargo	Targeted Host	Phage Feature	References
Antibiofilm	CPB0329	*Dispersin B*	*Klebsiella pneumoniae*	Replicative phage particles	[27]
Antimicrobial agent	T7	Colicin E1 and Colicin M	*Escherichia coli*	Replicative phage particles	[39]
Antimicrobial agent	T7	Colicin E1	*Escherichia coli*	Non-replicative particles *	[40]
Antimicrobial agent	T-even like phages	Cas (type I-E)	Pathogenic *Escherichia coli*	Replicative phage particles	[25]
Bacteria detection	T4	*LacZα*	*Escherichia coli*	Replicative phage particles	[43]
Bacteria detection	T7	NanoLuc luciferse	*Escherichia coli*	Replicative phage particles	[32]
Bacteria detection	PhiV10	*luxCDABE*	*Escherichia coli* (Food samples)	Replicative phage particles	[30]
Bacteria detection	E2, E4, EfS3, EfS7, K1and K4	NanoLuc luciferse	*Escherichia coli*, *Enterococcus* spp., and *Klebsiella* spp. (Urine samples)	Replicative phage particles	[29]
Bacteria detection	vB_Eco4M-7	HiBiT	STEC	Replicative phage particles	[31]
Bacteria genetic engineering	λ	Base editor dCas9	*Escherichia coli* (β-lactamase)	Non-replicative particles	[22]
Delivery of antimicrobial peptide	Sb-1	Nisin	MRSA	Phage structural components (Tail)	[42]
Drug delivery system for mammalian gut	T4	Serpine *B1a*, Chaperone protein *clpB*	Nonpathogenic *Escherichia coli*	Replicative phage particles	[33]
Gene delivery system for the human cells	T4	Gene editing, in situ protein expression and others	-	Phage structural components (Head)	[41]
Selective antimicrobial agent	λ	Cas12a	STEC	Non-replicative particles	[23]
Selective antimicrobial agent	P1	Cas9	*Escherichia coli*	Non-replicative particles	[44]
Selective antimicrobial agent	Phi80, M13, 80α and Tan2	Cas13a	*Escherichia coli* and *Staphylococcus aureus*	Non-replicative particles	[36,37,38]
Selective antimicrobial agent	P2	Cas9	*STEC* and *Shigella flexneri*	Non-replicative particles	[35]

*: The term non-replicative particles is used to distinguish between phage capsids and engineered lytic phages. STEC: Shiga toxin-producing *Escherichia coli*, MRSA: methicillin-resistant *Staphylococcus aureus*.

**Table 3:** Representative Engineered Phages and Associated In-vivo and In-vitro Engineering Strategies.

Phage General Information.		Phage Engineering Method.	In-Vivo Engineering Conditions.		In-Vitro (Synthetic) Engineering Conditions.					Engineering Purpose.		Reference
Phage Host.	Phage Name.		Recombineering Genes.	Counter Selection Method.	Assembly Method.	Rebooting Method.	Rebooting Host.	Intermediate Host.	Phage Genome Size Kbp.	Modification.	Purpose.	
*Escherichia coli*	Phage α15	In-vivo	-.	-	-	-	-	-	-	Gene replacement of ~7 kp	Load Cas (type I-E) and gene for Tsx-binding adhesin	[25]
*Escherichia coli*	T7	In-vivo	Flippase	Induced Phenotype *^1^	-	-	BW25113ΔtrxA	-	-	Gene replacement	Tail fiber modification	[116]
*Escherichia coli*	T4	In-vivo	-	Induced Phenotype *^2^	-	-	-	-	-	Gene replacement	In situ protein expression within mammalian cells	[33]
*Escherichia coli*	T4	In-vivo	-	CRISPR/Cas9	-	-	-	-	-	NanoLuc luciferase.	Reporter gene	[80]
*Escherichia coli*	T4	In-vivo *^3^	-	CRISPR/Cas9 or Cas12	-	-	-	-	-	Eliminate phage DNA packaging to create an empty head.	Load various cargoes to human cells	[41]
*Escherichia coli*	T3, T7, and T5	In-vivo	λ-red	CRISPR/Cas9	-	-	-	-	-	Point substitutions, insertions, or deletions.	Tail fiber modification	[98]
*Escherichia coli*	P1	In-vivo	λ-red	Selection Marker	-	-	-	-	-	Deletion of packaging region Δpac of plasmid phage P1	Phage capsid construction	[44]
*Escherichia coli*	λ.	In-vivo	λ-red	CRISPR/Cas9	-	-	-	-	-	Deletion	Tail fiber modification	[22]
*Klebsiella pneumonia.*	T7 family and non-family *Klebsiella pneumoniae* phages.	In-vivo	λ-red	CRISPR/Cas9	-	In-vivo	*Escherichia coli* DH10B	Yes	41 to 46	Either gene replacement of a non-essential ligase gene with dispersin B (DspB) or just gene insertion of the mentioned gene DspB	Distribute biofilm	[27]
*Anabaena*	Cyanophage A-1(L) and A-4(L)	In-vivo	-	CRISPR/Cas12a	-	-	-	-	-	Deletion	Minimize genome reduction of 2.4 kbp	[132]
*Pseudomonas aeruginosa*	-.	In-vivo	λ-red	CRISPR/Cas12a	-	-	-	-	-	Deletion	15 kbp deletion	[163]
*Pseudomonas aeruginosa.*	KZ	In-vivo	-	CRISPR/Cas13a and acrVIA1	-	-	-	-	-	Insertion, deletion and fluorescent tagging	-	[137]
*Escherichia coli.*	T4, T7 and EdH4	In-vivo.	-	CRISPR/Cas13a	-	-	-	-	-	Multi gene deletion and single base modification	-	[136]
*Escherichia coli.*	T7	In-vivo.	-	Recombitrons	-	-	-	-	-	Amino acid substitutions in gp17 gene	Expand host range	[82]
*Escherichia coli.*	T7	Synthetic	-	-	NEBuilder HiFi DNA	In-vivo	*Escherichia coli* 10G	No	39.937 + 0.977	Insertion of NanoLuc luciferase	Reporter gene	[32]
*Escherichia coli, Klebsiella and Yersinia.*	T7 family	Synthetic	-	-	YAC	In-vivo	*Escherichia cloni* 10G	Yes	37 to 45	Modify Tail fiber	Expand host range	[76]
*Escherichia coli.*	T7	Synthetic	-	-	Exonuclease only	TXTL	-	-	39	Modify Tail fiber	Expand host range	[164]
*Salmonella.*	P22	Synthetic	-	-	Gibson	In-vivo	*Salmonella Typhimurium* strain LT2	-	-	Deletion of lytic cycle repressor *c2*	Modify lifestyle	[78]
*Mycobacterium.*	D29	Synthetic	-	-	Gibson	In-vivo	*M. smegmatis* mc2155	-	-	Gene replacement and insertion of NanoLuc luciferase gene	Reporter	
*Escherichia coli*	T7	Synthetic	-	-	Gibson	TXTL	*-*	-	-	Gene replacement and insertion of LacZ operon	Reporter	
*Salmonella*	SP6	Synthetic	-	-	Gibson	In-vivo	*Salmonella Typhimurium* strain LT2	-	-	Deletion of phage head	Biocontained Phages	

Notes and Abbreviations: *^1^ The trxA gene encoding thioredoxin was inserted into the T7 phage genome. The engineered phage was propagated using an *E. coli* BW25113Δ*trxA* host strain to enable selection of recombinants. *^2^ Genes of interest were inserted into the ac gene locus of T4 phage; deactivation of this gene confers resistance to acridine dye, and phage proliferation with this substance will affect the wild-type T4 but not the engineered variant [33,165]. *^3^ T4 phage capsids lacking genomic DNA (empty heads) were developed using amber mutants in neck and tail genes (amber10 and amber13), which introduce premature stop codons. When propagated in non-suppressor *E. coli* strains, these mutants prevent the production of complete virions. Pac: Phage packaging site. acrVIA1: Anti-CRISPR gene used to suppress Cas13a activity against the engineered phage. NEBuilder: A high-efficiency variant of Gibson assembly optimized for complex or multiple-fragment DNA constructs.

## Data Availability

Data are contained within the article.
